# Assessment of Neurodevelopmental Outcomes Using the Developmental Assessment Scale for Indian Infants and Growth Patterns Among High-Risk Infants From a Special Newborn Care Unit in Northern India: A Longitudinal Study

**DOI:** 10.7759/cureus.107183

**Published:** 2026-04-16

**Authors:** Mangla Sood, Shagun Kaushal, Rajender Thakur, Ishaan Sood, Anupam Singh

**Affiliations:** 1 Pediatrics, Indira Gandhi Medical College, Shimla, IND; 2 General Practice, Indira Gandhi Medical College, Shimla, IND; 3 Clinical Psychology, Indira Gandhi Medical College, Shimla, IND

**Keywords:** anthropometry, development assessment scale, growth and development, meningitis, shock

## Abstract

Objective: To assess neurodevelopmental outcomes and growth in high-risk infants post discharge from a level 3 newborn unit.

Methodology: Eligible high-risk infants were enrolled in this longitudinal follow-up study, and anthropometric measurements were interpreted using the WHO Anthro software (WHO, Geneva, Switzerland) at three, six, nine, and 12 months corrected age (CA). Neurodevelopment assessment was done using the Developmental Assessment Scale for Indian Infants (DASII) at six- and 12-month CA. Logistic regression analysis was used to identify risk factors associated with developmental delays.

Result: Among 296 high-risk infants, 194 completed 12-month follow-up; seven died (2.4%), and 95 were lost to follow-up. Motor delay affected 29 (12.6%) at six months and 15 (7.7%) at 12 months; corresponding figures for mental delay were nine (4%) and seven (3.6%). At 12 months, the strongest predictors of motor delay were shock (OR: 78.1; 95% CI: 26.6, 228), hypoxic-ischemic encephalopathy (HIE) (OR: 65.5; 95% CI: 14.1, 304), and meningitis (OR: 25.9; 95% CI: 8.2, 82.1). Significant predictors for mental delay included HIE (OR: 92.6; 95% CI: 12, 113.6), shock (OR: 79.6; 95% CI: 23.3, 271.7), meningitis (OR: 42; 95% CI: 9.3, 188), and ventilator support >24 hours (OR: 13; 95% CI: 6.4, 26.6). Other factors like birth weight <1000 grams (OR: 5.7; 95% CI: 2.0, 15.9) and sepsis (OR: 2.6; 95% CI: 1.4, 4.9) also remain significant risks throughout the first year. Growth faltering was greatest for weight/length-for-age Z-scores at three months and head circumference at nine months.

Conclusion: Neurodevelopmental delays declined by 12 months but persisted in a vulnerable subset, with shock, HIE, and meningitis emerging as the strongest predictors. Growth faltering was most evident in early weight/length indices and later in head circumference.

## Introduction

The establishment and rapid expansion of special newborn care units (SNCUs) across India have led to a commendable decline in neonatal mortality. Improved access to specialized intensive care has significantly increased survival rates among premature, very low birth weight (VLBW), and critically ill infants [1]. However, this life-saving success has introduced a new clinical challenge. We are now caring for a growing population of high-risk SNCU graduates who remain uniquely vulnerable to long-term health issues. Because these fragile infants often endure prolonged intensive care, systemic inflammatory insults, and early nutritional deficits, they face a substantially higher risk of extrauterine growth restriction (EUGR) and subsequent neurodevelopmental delays [2].

Despite the growing size of this high-risk cohort, comprehensive, long-term follow-up data in India are still severely lacking. This makes it difficult to understand the true epidemiological burden, allocate healthcare resources effectively, or provide families with the evidence-based counseling they need [2,3]. Of the 1.2 million Indian infants currently living with moderate to severe disabilities, approximately 40% have a documented history of perinatal complications [4]. This underscores the critical necessity for the systematic monitoring of high-risk infants where early intervention can alter developmental trajectories [5].

Furthermore, while the global literature offers extensive data on neurodevelopmental outcomes, much of it relies heavily on Western assessment models. Preliminary regional studies using various scales suggest that a substantial proportion of these high-risk infants experience developmental setbacks, with delay rates reported at 31.6% at 12 months of corrected age in some cohorts [6] and reaching up to 50% in others [5]. To accurately evaluate the local population, there is a critical need for prospective data from culturally and socio-economically validated tools, such as the Developmental Assessment Scale for Indian Infants (DASII) [7].

To help bridge these gaps, we conducted this prospective study to track the longitudinal trajectories of high-risk infants discharged from an SNCU at a tertiary teaching hospital in Northern India. The primary objectives were to identify deviations in somatic catch-up growth and determine the prevalence of neurodevelopmental delays using the DASII. The secondary objective was to identify the specific perinatal risk factors contributing to these delays. Ultimately, defining these predictors is vital for optimizing early intervention protocols and enhancing survivorship quality.

## Materials and methods

A prospective longitudinal study was conducted from June 2023 to December 2024 in a 24-bedded level 3 newborn unit staffed by pediatricians and postgraduate students, with two or three staff nurses available on every shift, with the facility to resuscitate, provide surfactant, provide respiratory support, and provide assisted ventilation. The neonates were managed according to the National Neonatology Forum (NNF) guidelines [8].

The study included high-risk infants discharged alive with birth weight less than 1500 grams, and/or gestation below 32 weeks, or higher weight and gestation with unstable clinical course (birth asphyxia, seizures, sepsis, shock, respiratory distress, double volume exchange transfusion, symptomatic hypoglycemia, etc.). Neonates with birth defects or genetic abnormalities or requiring surgery for congenital abnormalities were excluded. The study protocol was approved by the institutional ethics committee, and written informed consent was obtained from the parent/caregiver. The reporting of this study conforms to the Strengthening the Reporting of Observational Studies in Epidemiology (STROBE) statement guidelines.

As the study was time-bound, no formal sample size calculation was performed; instead, all eligible infants discharged between June 2023 and November 2023 whose parents consented were enrolled.

A pre-structured form was used to record the relevant antepartum, intrapartum, and birth details from maternal case records. Gestational age was determined based on the first-trimester scan, if available, or by the new Ballard score. Neonatal data, including gender, birth weight, resuscitation details, and Apgar scores, were recorded. Comorbidities observed during the SNCU stay were also recorded. Enrolled infants were evaluated at corrected age (CA) of three, six, nine, and 12 months with a time tolerance limit of ± 7 days. If an appointment was missed, every effort was made to contact caregivers telephonically and remind them of the scheduled visit.

At every follow-up visit, the neonates were assessed for anthropometry, vision and hearing, neurological examination (muscle tone and angles as described by Amiel-Tison, reflexes, movements), and developmental assessment. Screening for retinopathy of prematurity (ROP) was done and classified according to the International Classification of Retinopathy of Prematurity (ICROP) [9]. Hearing screening was done by oto-acoustic emission (OAE) (easyScreen ABR+OAE, Maico Diagnostics, Berlin, Germany) at 34 weeks of gestation or before discharge, whichever was later. Infants who failed on OAE were further assessed by brainstem evoked response auditory (BERA) before three months of age. Cranial ultrasonography was performed as per the unit protocol.

A single trained researcher conducted all neurological and anthropometric evaluations. Weight, length, and head circumference were measured using a digital infant weighing scale (Seca 334, Seca, Hamburg, Germany), infantometer (Seca 417), and non-stretchable tape, respectively. All equipment was calibrated, and standard precautions were taken. Growth was assessed by measuring weight-for-age, length-for-age, and head circumference-for-age, and plotted on Fenton charts until 40 weeks of CA, after which the WHO child growth standards were used, and Z-scores were computed using WHO Anthro software (WHO, Geneva, Switzerland) [10].

The neurodevelopmental assessment was conducted at six months and one-year CA using the DASII, an open-access Indian adaptation of the Bayley Scales of Infant Development, validated for children up to 30 months [7]. Assessment was carried out by a trained psychologist (AS) and covered two domains, motor and mental, and scored according to the DASII manual. Neurodevelopmental outcome was defined as the proportion of children with developmental delay as indicated by a DASII score below 70. Those with a major neurodevelopmental abnormality were enrolled in the developmental neurology clinic, and appropriate therapy was initiated [11].

Statistical analysis

Statistical analyses were performed using Stata version 14 (StataCorp LLC, College Station, TX). Descriptive statistics were used to examine associations between study variables and outcomes. Group comparisons employed independent t-tests, paired t-tests, repeated-measures ANOVA, and chi-square tests, as appropriate. Logistic regression identified variables associated with DASII motor and mental scores <70, and the correlation coefficient quantified associations with growth. A two-sided P < 0.05 was considered statistically significant.

## Results

**Figure 1:**
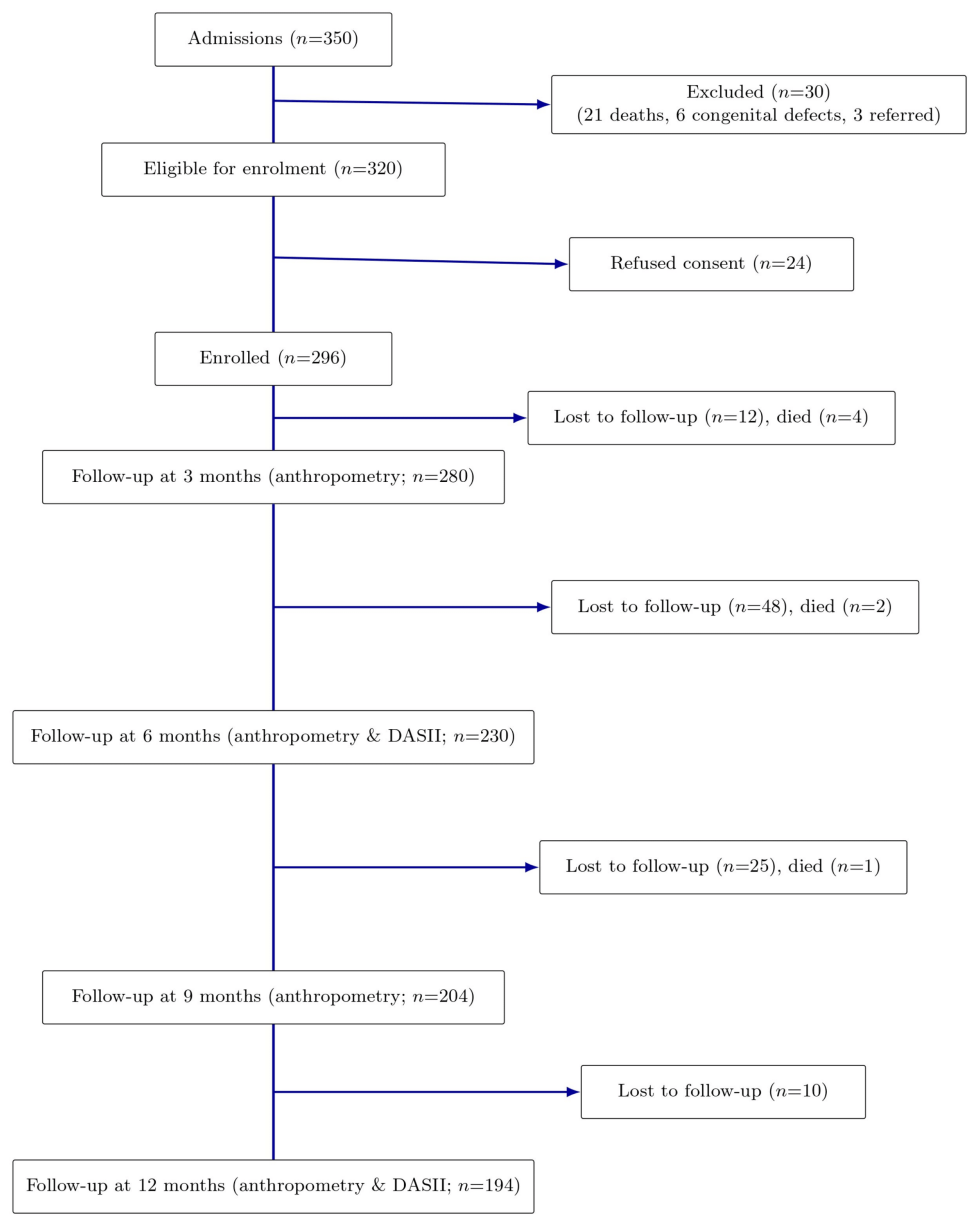
Flow chart of participants. DASII: Developmental Assessment Scale for Indian Infants.

Of 350 eligible neonates, 296 were enrolled, with seven deaths and an additional 95 infants being lost to follow-up for various reasons (Figure 1).

The median (q1, q3) birth weight was 1317 (800, 2400) grams, and the mean (SD, range) gestation was 30.2 (3.2; 27 to 35) weeks; 110 were born through cesarean section. The median (range) SNCU stay was 18 (6-86) days. The morbidity profile of the cohort is described in Table 1.

**Table 1:** Baseline variables and clinical outcome at discharge of enrolled infants (n = 296). CPAP: continuous positive airway pressure; hsPDA: hemodynamically significant patent ductus arteriosus; ROP: retinopathy of prematurity. ^a^ Sepsis was diagnosed based on criteria specified by the National Neonatology Forum, and includes culture-positive and culture-negative sepsis.

Variable	n (%)
Male gender	180 (60.8)
Birth weight (g)	
<1000	24 (8)
1000-1500	200 (68)
≥1500	72 (24)
Appropriate-for-date	186 (62.8)
Small-for-date	107 (36.1)
Large-for-date	3 (0.1)
Gestation (weeks)	
<32	236 (79.7)
≥32	60 (20.3)
Respiratory distress syndrome	125 (42.2)
Received surfactant	25 (8.4)
Received CPAP for >24 hours	85 (28.7)
Received mechanical ventilation for >24 hours	40 (13.5)
Hypoxic-ischemic encephalopathy (HIE)	16 (2.7)
HIE stage II	14
HIE stage III	2
Sepsis^a^	128 (43.2)
Meconium aspiration syndrome (MAS)	14 (4.7)
Pneumonia	38 (12.8)
Hypoglycemia	34 (11.5)
Meningitis	16 (5.4)
Neonatal jaundice requiring exchange transfusion	8 (2.7)
Neonatal seizures	22 (7.4)
Intraventricular hemorrhage	48 (16.2)
Grade 2	38
Grade 3	10
Bronchopulmonary dysplasia	14 (4.7)
hsPDA	12 (4)
ROP requiring laser treatment	18 (6)
Hearing impairment	4 (1.4)
Periventricular leukomalacia (on follow-up ultrasound)	12 (4)

The serial anthropometric measurements at different time points are presented in Table 2. At six months CA, mean (SD) DASII motor and mental scores were 78.6 (10.2) and 82.7 (10.6), with 29 (12.6%) and nine (4%) scoring <70, respectively. At 12 months CA, motor and mental scores rose to 80.8 (10.2) and 83.7 (9.3), with 15 (7.7%) and seven (3.6%) scoring <70, respectively (Table 2). Among those identified with neurodevelopmental delays at six months, two were lost to follow-up before 12-month CA.

**Table 2:** Anthropometry and DASII scores at follow-up. Data are expressed as mean (SD). HC: head circumference; HCFA: head circumference-for-age; LFA: length-for-age; WFA: weight-for-age; DASII: Developmental Assessment Scale for Indian Infants.

Age	Weight (g)	WFA Z-score	Length (cm)	LFA Z-score	HC (cm)	HCFA Z-score	DASII motor score	DASII mental score
At discharge (n=296)	1417 (598.5)	-2.8 (1.2)	48.6 (4.8)	-1.5 (1.3)	32.7 (1.1)	-1.6 (1.1)	Not analyzed	Not analyzed
3 months (n=280)	2698.7 (812.3)	-3.1 (1.3)	52.4 (5.3)	-2.8 (1.3)	34.9 (2.4)	-2.5 (1.3)	Not analyzed	Not analyzed
6 months (n=230)	4528.6 (1313.2)	-2.4 (1.1)	64.7 (4.1)	-2.1 (1.5)	40.4 (2.1)	-2.4 (1.5)	78.6 (10.2)	82.7 (10.6)
9 months (n=204)	7888.7 (763.7)	-2.1 (0.8)	68.6 (4.2)	-1.9 (1.2)	42.7 (1.6)	-2.1 (1.2)	Not analyzed	Not analyzed
12 months (n=194)	8243.3 (1086.7)	-1.9 (1.08)	72.4 (3.24)	-1.3 (0.8)	44.3 (1.9)	-2.1 (1.3)	80.8 (10.2)	83.7 (9.3)

At six and 12 months, the presence of shock, hypoxic-ischemic encephalopathy (HIE), meningitis, mechanical ventilation >24 hours, extremely low birth weight (ELBW, <1000 g), neonatal jaundice requiring exchange transfusion, intraventricular hemorrhage (IVH), sepsis, respiratory distress syndrome (RDS), and hypoglycemia at birth were associated with motor delay. Additionally, the need for prolonged continuous positive airway pressure (CPAP) was associated with motor delay at 12 months.

For motor delay (Table 3), the risk profile closely mirrored that for mental delay, as shown in Table 4. Gestational age <32 weeks by itself, hemodynamically significant patent ductus arteriosus (hsPDA), and apnea were not associated with motor or mental delay.

**Table 3:** Predictors for motor delay in high-risk infants. RDS: retinopathy of prematurity; CI: confidence interval; CPAP: continuous positive airway pressure; DVET: double volume exchange transfusion; HIE: hypoxic ischemic encephalopathy; hsPDA: hemodynamically significant patent ductus arteriosus; IVH: intraventricular hemorrhage; NNJ: neonatal jaundice; OR: odds ratio.

Predictors	OR (95%CI)	P-value	OR (95% CI)	P-value
	Motor delay at 6 months		Motor delay at 12 months	
Gestation < 32 weeks	1.6 (0.5, 4.9)	0.366	1.5 (0.6, 3.9)	0.453
Extremely low birth weight	7.6 (1.7, 33.6)	0.007	6.7 (2.0, 21.5)	0.008
RDS	3.4 (1.5, 7.8)	0.033	3.5 (1.7, 7.2)	0.002
CPAP > 24 hours	1.8 (0.8, 4.1)	0.113	2.3 (1.1, 4.7)	0.032
Ventilation > 24 hours	8.6 (3.7, 19.8)	<0.001	11.9 (5.2, 23.9)	<0.001
HIE stage II/III	32.1 (10.0, 102.1)	<0.001	65.5 (14.1, 304.5)	<0.001
Hypoglycemia	3.5 (1.4, 8.7)	0.007	3.1 (1.4, 7.3)	0.024
IVH grade 2/3	4.5 (2.0, 10.2)	0.003	5.8 (2.7, 12.0)	<0.001
hsPDA	1.9 (0.3, 9.3)	0.423	2.3 (0.6, 8.6)	0.259
NNJ with DVET	6 (1.3, 26.7)	0.018	4.3 (0.9, 20.1)	0.043
Sepsis	3.2 (1.4, 7.4)	0.005	2.8 (1.3, 5.6)	0.012
Meningitis	16.7 (5.6, 49.6)	<0.001	25.9 (8.2, 82.1)	<0.001
Apnea	1.4 (0.6, 3.2)	0.321	1.2 (0.6, 2.4)	0.593
Shock	57.7 (20.1, 165.6)	<0.001	78.1 (26.6, 228.5)	<0.001

**Table 4:** Predictors for mental delay in high-risk infants. RDS: retinopathy of prematurity; CI: confidence interval; CPAP: continuous positive airway pressure; DVET: double volume exchange transfusion; HIE: hypoxic ischemic encephalopathy; hsPDA: hemodynamically significant patent ductus arteriosus; IVH: intraventricular hemorrhage; NNJ: neonatal jaundice; OR: odds ratio.

Predictors	OR (95% CI)	P-value	OR (95% CI)	P-value
	Mental delay at 6 months		Mental delay at 12 months	
Gestation < 32 weeks	1.4 (0.5, 3.5)	0.327	1.34 (0.6, 3.0)	0.479
Extremely low birth weight	4.5 (1.5, 13.8)	0.001	5.7 (2.0, 15.9)	0.001
RDS	3.0 (1.5, 6.2)	0.001	3.1 (1.6, 5.8)	0.004
CPAP > 24 hours	2.2 (1.1, 4.5)	0.019	2.2 (1.1, 4.1)	0.013
Ventilation > 24 hours	9.6 (4.5, 20.5)	<0.001	13 (6.4, 26.5)	<0.001
HIE stage II/III	44.2 (12.0, 162.2)	<0.001	92.6 (12.0, 713.6)	<0.001
Hypoglycemia	2.6 (1.1, 6.2)	0.006	2.8 (1.3, 6.1)	0.006
IVH grade 2/3	5.0 (2.4, 10.7)	0.001	6.1 (3.1, 12.0)	<0.001
hsPDA	2.1 (0.6, 8.0)	0.203	2.1 (0.6, 6.8)	0.191
NNJ with DVET	4.7 (1.1, 21.7)	0.062	4.6 (1.1, 19.2)	0.034
Sepsis	2.5 (1.2, 5.2)	0.005	2.6 (1.4, 4.9)	0.002
Meningitis	20.6 (6.8, 62.1)	0.001	42 (9.3, 188.1)	<0.001
Apnea	1.1 (0.6, 2.4)	0.584	1.2 (0.6, 2.2)	0.537
Shock	72 (24.6, 210.1)	0.001	79.6 (23.3, 271.6)	<0.001

## Discussion

We followed up 194 high-risk infants and assessed their growth and neurodevelopmental outcomes at one-year CA. All seven deaths happened at home, and the exact cause was not known. At six months CA, 12.6% and 4% of the study cohort had motor and mental delay (DASII <70), and these proportions reduced to 7.7% and 3.6% at 12 months CA.

As in earlier studies, anthropometry and gestational age were shown to be significantly correlated [12,13]. The mean z-scores for weight and length were below and farthest from the population median at three months CA, and subsequently improved. However, head circumference z-scores improved only after nine months CA. This may suggest that somatic growth in low birth weight infants recovers earlier than neural growth.

Damage to the developing brain manifests with motor disability, cognitive impairment, and sensory impairment [10,14]. Established risk factors include low birth weight and gestational age, multiple births, non-use of antenatal corticosteroid, sepsis and meningitis, hypoglycemia and metabolic cause, periventricular leukomalacia, and IVH [15,16]. Small sample sizes in a few subgroups (neonatal jaundice, HIE, meningitis), however, resulted in wide confidence intervals.

The mean (SD) motor and mental scores using DASII in the current cohort were 80.8 (10.2) and 83.7 (9.3), respectively, at 12 months CA, which are much higher than those reported from another study from India [17]. In the current cohort of intramural high-risk neonates, motor delay was identified in 7.7% and mental delay in 3.6% of infants at 12 months CA, which was also much lower than that reported previously [16,18,19], although comparable figures were reported in a retrospective study from western India [20]. The observed differences across studies likely reflect variability in the standards of neonatal care across centers, as well as differences in the population characteristics, referral patterns, and disparities in access to maternal and perinatal services, all of which directly influence neurodevelopmental outcomes.

Adherence to the NNF protocols, regular in-service training of staff on neuroprotective and development supportive care, and early family participation in daily care activities in the current cohort contributed to favorable outcomes.

Damage to the developing brain manifests with motor disability, cognitive impairment, and sensory impairment [10,14]. Severe neonatal complications cause motor and cognitive delays, reflecting shared pathways of neurological injury and vulnerability. The current study reiterates that systemic insults and direct neurological injuries, particularly shock, HIE, and meningitis, contribute to increased risk of adverse neurodevelopmental outcomes consistent with existing evidence [15,16,21]. Conversely, gestational age <32 weeks, apnea, and hsPDA did not demonstrate statistically significant associations with motor or mental delay at any time point, suggesting that these factors, in isolation, may be less predictive.

Although prematurity has long been regarded as a major risk factor for adverse neurodevelopment [15], in the current cohort, ELBW emerged as a significant predictor. In contrast, gestational age <32 weeks alone did not affect neurodevelopmental outcome significantly, but specific morbidities associated with prematurity led to physiological instability and direct neurological injury [22-25]. While the decline in the rates of motor and mental delay between six and 12 months is encouraging, it is crucial to recognize that this short follow-up period likely captures early developmental catch-up rather than the achievement of definitively normal long-term growth. Continuous surveillance is required to ensure these trajectories are maintained.

The main strength of the current study is a comprehensive documentation of morbidities, mortality, and outcomes in low-birth-weight babies from resource-constrained settings. The limitations include a short 12-month follow-up period and significant attrition, with 95 infants lost to follow-up despite repeated parental counseling. The risk factors identified in the study represent temporal associations rather than definitive causal relationships. In addition, this study, without a predefined sample size and with more than 80% of high-risk neonates born before 32 weeks, may have limited the power to detect all risk factors for growth and developmental delay of high-risk late preterm and term infants. We propose a longer follow-up, improved communication with parents, and telephonic or virtual follow-up to enhance retention, enable earlier detection of developmental disorders, and guide timely interventions. A large multicentric study will better define growth and neurodevelopmental trajectories in this population.

## Conclusions

The implications of these findings are significant for clinical practice and provide data to support and develop regional guidelines for care and neurohabilitation of this vulnerable group. The variables identified here can be used to stratify infants into risk categories upon discharge from the neonatal unit, enabling timely, targeted neurohabilitation therapy that can help reduce the severity of delays and maximize developmental potential, particularly in resource-limited settings.
